# Antioxidants (selenium and garlic) alleviated the adverse effects of tramadol on the reproductive system and oxidative stress markers in male rabbits

**DOI:** 10.1038/s41598-022-16862-4

**Published:** 2022-08-17

**Authors:** Salah A. Sheweita, Yassmin A. El-dafrawi, Osama A. El-ghalid, Alaa A. Ghoneim, Ahmed Wahid

**Affiliations:** 1 Department of Clinical Biochemistry, Faculty of Medicine, King Khalid University, P.O.Box: 960, Abha, 61421 Kingdom of Saudi Arabia; 2 Department of Biotechnology, Institute of Graduate Studies and Research, University of Alexandria, Alexandria, Egypt; 3 Poultry Physiology Department, Faculty of Agriculture, University of Alexandria, Alexandria, Egypt; 4 Department of Anaesthesia and Pain Management, Medical Research Institute, Alexandra University, Alexandria, Egypt; 5 Department of Pharmaceutical Biochemistry, Faculty of Pharmacy, Alexandria University, Alexandria, Egypt

**Keywords:** Biochemistry, Cell biology, Chemical biology, Endocrinology, Health care, Medical research

## Abstract

Tramadol has been used by millions of patients as an analgesic drug to relief the severe pain caused by cancers and other diseases. The current study aimed to investigate the protective effects of antioxidants (garlic and selenium) against the toxic effects of tramadol on semen characteristics, steroid hormones, the protein expressions of different cytochrome P450 isozymes [CYP 21A2, CYP 19, and 11A1], and on antioxidant enzyme activities in testes of rabbits. Western immunoblotting, spectrophotometric, and histological methods were used in this study. Tramadol (1.5 mg/kg body weight) was administered orally to male rabbits for up to three months (three times/week), and after pretreatment of rabbits with garlic (800 mg/kg) and/or selenium (1 mg/kg body weight) by 2 h. The present study showed that motilities, semen volumes, morphologies, sperm counts, testosterone, and estrogen levels were significantly decreased after 4, 8, and 12 weeks of tramadol treatment. In addition, the protein expressions of CYP 21A2, CYP 19, and 11A1 were down-regulated in the testes of the tramadol-treated rabbits. On the other hand, pretreatment of rabbits with garlic, selenium, and/or garlic-selenium for 2 h before administration of tramadol restored the downregulated CYP 21A2 and 11A1 to their normal levels after 12 weeks of tramadol treatment. Activities of antioxidant enzymes including glutathione reductase, glutathione peroxidase, glutathione S-transferase, catalase, superoxide dismutase, and levels of glutathione were inhibited in the testes of tramadol-treated rabbits. On the other hand, free radical levels were significantly increased in the testes of tramadol-treated rabbits for 12 weeks. Interestingly, such changes in the activities of antioxidant enzymes as well as free radical levels caused by tramadol were restored to their normal levels in the rabbits pretreated with either selenium, garlic, and/or their combination. Histopathological investigations showed that tramadol caused substantial vacuolization with the presence of damaged immature spermatozoid in the testes. However, selenium and garlic treatments showed an increase in healthy sperm production with normal mitotic and meiotic divisions. The present study illustrated for the first time the mechanisms of low steroid hormone levels in the testes of tramadol-treated rabbits which could be due to the downregulation of CYPs proteins, induction of oxidative stress, and inhibition of antioxidant enzyme activities. In addition, the present data showed that such toxic effects of tramadol were attenuated and restored to their normal levels after pretreatment of rabbits with garlic, selenium, and/or their combination. This finding may pave the way for a new approach to reducing the toxicity of tramadol.

## Introduction

Numerous new analgesic drugs have been used to relieve mild to severe pain conditions^1^. Tramadol is a highly effective analgesic drug that is used to relieve acute and chronic pains through its binding to the μ-opioid receptor and inhibiting norepinephrine and serotonin uptake in neurons^2–4^. Tramadol is metabolically activated by CYP3A4 and CYP2D6 into a more powerful opioid analgesic metabolite M1^4–7^. Tramadol's opioid analgesic efficacy was affected by the CYP isozymes, because patients with low metabolizers converted less tramadol to the active M1 metabolite, whereas those with higher metabolic rates had the greatest analgesic effects. Furthermore, polymorphisms in the CYP2D6 gene increased hepatotoxicity by accumulating tramadol bioactive metabolite (M1)^8^. The M1 metabolite of tramadol is well known to cause severe toxic effects in different organs of rats and is mainly detoxified after its conjugation with glucuronic acid and sulfate^9,10^.

Tramadol has been shown to reduce sperm count, motility, and morphology while also increasing oxidative stress markers in rat testes^11,12^. Also, it caused testicular mitochondrial dysfunction by producing swelling of the testicular mitochondrial membrane. Furthermore, tramadol increased pro-apoptotic Bax expression as well as levels of tumor necrosis factor-, interleukin-1, and nuclear factor kappa B, while decreasing anti-apoptotic Bcl-2 expression and causing histological degenerative changes in the testes^11,12^. Also, tramadol lowered serum levels of luteinizing hormone (LH), follicle-stimulating hormone (FSH), and testosterone in rats and humans in another research^13,14^. Degenerative changes in the seminiferous tubules were also seen, and Leydig cells in the testes of rats had euchromatic nuclei and dilated smooth endoplasmic reticulum^13^.

Infertility is one of the major medical and social problems^15^. Infertility, which affects about 40% of all couples, is linked to different causes^16^. Unfortunately, many men with normal semen analysis findings are still infertile^13^. This means that standard semen analysis may not always provide complete diagnostic information In about 25% of partners, there is no actual cause for primary or secondary infertility, which is referred to as undiagnosed infertility^18,19^. Therefore, oxidative stress [OS] and reactive oxygen species (ROS) generation are two suggested causes behind the undiagnosed and unexplained infertility^20–22^. Therefore, the decrease in sperm function has been linked to oxidative stress, which happens when antioxidant enzyme activities are inhibited, and antioxidants are overwhelmed by oxidants^23–25^. It has been found that oxidative stress disrupted the function of sperms via generation of free radicals in the seminal fluid^26,27^. In addition, antioxidant enzymes including glutathione S-transferase, glutathione peroxidase, glutathione reductase, superoxide dismutase have been found to play an important role in scavenging free radicals in the seminal fluid, which consequently could improve the fertility in the human being^22^.

The biosynthesis of steroid hormones is mainly mediated by cytochrome P450 isozymes including CYP11A, CYP11B1, and CYP11B2 which are localized on mitochondrial membrane-bound proteins, whereas CYP17, CYP19, and CYP21 are localized in the endoplasmic reticulum (microsomal) membrane-bound proteins^28,29^. The steroidogenic acute regulatory protein (StAR) was responsible for transferring cholesterol from the outer mitochondrial membrane to the inner one, which hosts the cytochrome P450 side-chain cleavage (P450 SCC) enzyme^30^. P450scc converts cholesterol to pregnenolone, which is subsequently transported to the smooth endoplasmic reticulum, where it is converted to testosterone by CYP17, CYP21, 3-hydroxysteroid dehydrogenase (3-HSD), and 17-hydroxysteroid dehydrogenase (17-HSD)^31–35^. In the testes, the 17-HSD enzyme is almost expressed and is needed for testosterone biosynthesis^36^. It has been found that inhibition17-HSD causes male pseudo hermaphroditism^33^. CYP19 is working as the rate-limiting enzyme in changing androgens to estrogens. Therefore, CYP19 is a key enzyme in estrogens biosynthesis^38,39^.

Garlic and selenium have been shown in our previous research to protect the liver and other organs of rats against the harmful effects of toxic compounds^40–43^. To our knowledge, no previous study has looked at the mechanisms of low steroid hormone levels after tramadol treatment. Therefore, under the impact of tramadol alone or after pre-treatment of rabbits with garlic, selenium, and/or their combination, alterations in the protein expression of cytochrome P450 isozymes [CYP 21A2, CYP 19, and 11A1] involved in the steroidogenesis of steroid hormones were studied. Furthermore, changes in the antioxidant enzyme activities, semen properties, and the architecture of male rabbit testes were studied.

## Materials and methods

### Materials

ABCAM1 pharmaceuticals, UK, provided a Western blotting detection kit, primary anti-rabbit antibodies for CYP 21A2 [Cat no:67421-1-Ig], CYP 19 [Cat no.PA1-21398], CYP 11A1 [Cat no:MO-AB-07770Y], and secondary antibody-antirabbit-HRP [Cat no: ab6721]. Tramadol hydrochloride [ N02AX02] was obtained from Accord-UK Ltd, UK. Sigma Chemical Company (Saint Louis, USA) provided all the other chemicals.

### Animals

Twenty-five male New Zealand white rabbits aged seven months and weighing 2.5–3.5 kg at the beginning of the experiment were used. Rabbits were purchased from the Breeding Rabbit Section, Poultry Research Center, Faculty of Agriculture, Alexandria University, Egypt. The experimental design and technique, which meet the requirements of the National Institute of Health, were authorized by the local committee of animal care at Graduate Studies and Research, Alexandria University, Egypt. In addition, the committee approved all used methods following the relevant guidelines and regulations of the published papers. We confirm that the present study is reported following the “ARRIVE guidelines” statement. The rabbits were housed in stainless steel-bottomed wire cages in a well-ventilated animal house with a temperature of 22 °C, relative humidity of 40–60%, a 12-h light/dark cycle, and free access to a pellet meal. After one week of acclimatization, the rabbits were divided into five equal groups [five rabbits/each]. Five rabbits in each group were chosen according to the study of Almodin et al.^43^.

### Research design

Group I: Rabbits received 0.5 ml saline/kg body weight by oral gavage three times a week for 12 weeks. Group II, rabbits received 1.5 mg/kg tramadol dissolved in saline by oral gavage three times per week for 12 weeks. Rabbits in groups III, IV, and V were pretreated with sodium selenite (1 mg/kg), allium sativum garlic extract (800 mg/kg), and garlic (800 mg/kg) plus sodium selenite (1 mg/kg) for 2 h before administration of tramadol three times per week for 12 weeks. Tramadol, garlic, and selenium doses have been chosen based on previous studies^10,44,45^. A human-like tramadol dosage was used.

### Methods

#### Semen analysis

Ejaculations were collected in a clean glass wide container using an artificial vagina every 4 weeks during the 12 weeks of the study. The specimen was incubated at 37 °C during the liquefaction of the semen. Semen analysis was started after liquefaction within 30–60 min of semen collection to prohibit dehydration or changes in the temperature that can affect semen quality.

#### Sperm motility

The sperm motility test was assayed using the method of Atashfaraz et al*.*^46^. 10 µl of the sperm suspension was put on a clean, pre-warmed slide at 27 °C. A coverslip was used to protect it. Then, using a light microscope (Leica DM 750) on a stage heated to 37 °C, slides were observed at 100 times magnification.

#### Sperm count

The sperm count was estimated using the method of Freund and Carol^47^. After collecting the semen, 50 µl of semen [ejaculate] was diluted with 2 ml normal saline and prewarmed to 37 °C. After that, the spermatozoa were diluted and suspended in regular saline. 200 µl of the suspension was pipetted into the two chambers of a Neubauer hemocytometer using a Pasteur pipette. This was accomplished by contacting the coverslip's edge and enabling capillary action to fill each of the chambers^47^. The spermatozoa were then counted under a microscope (Leica DM 750).

#### Sperm morphology

To examine sperm morphology, we put a drop of the sperm solution prepared for sperm count on a glass slide. After that, the suspension was stained with 1% eosin, air dried, and examined at a magnification of 400 times under the light microscope^48^. In each animal, we computed anomalies in the head, middle piece, and tail of spermatozoa.

#### Blood samples

After every 4 weeks of treatment throughout the whole period of the study, about 3 ml of blood withdrawn from the marginal ear veins of each rabbit from each group and placed in clean tubes with a clot activator or gel for serum separation. The clear serum was isolated from coagulated blood samples and stored at − 80 °C after centrifugation at 3000×*g* for 15 min.

#### Hormonal assays

The concentrations of testosterone and estrogen measured in the serum using solid-phase enzyme immunoassay (ELISA) kits (Diagnostics Biochem, Canada Inc. AIA -360). 20 µl of serum was added to a cell containing magnetic beads coated with immobilized antibodies/antigens and incubated to start the immunological response. When the incubation period has ended, the excess mixture rinsed away, the substrate and fluorescence reagents added, and the reaction mixture incubated. After the incubation time, the fluorescence intensity measured with a spectrophotofluorometer at a wavelength of 940 nm.

#### Preparation of the microsomal fraction

At the end of the 12th week, rabbits fasted for 12 h, and after that received intramuscular injections of xylazine for anesthesia (10 mg/kg), and then sacrificed^49^. The testes were removed, rinsed in a cold 0.1 M potassium phosphate buffer (PH 7.3), allowed to dry, and weighed. A Teflon piston homogenizer was used to homogenize the testes in 3 volumes of 0.1 M phosphate buffer (pH 7.3) at 4 °C. To eliminate nuclei and cell debris, the testes' homogenates were centrifuged for 20 min at 4° C. at 12,000×*g*. 1.5 ml of supernatant was stored at − 80 °C for biochemical analysis. The remaining supernatant was ultra-centrifuged at 105,000×*g* for 60 min at 4 °C to sediment microsomal pellets. Finally, the microsomal pellets containing cytochrome P450 isozymes were suspended in 0.1 M potassium phosphate buffer (PH 7.3) and frozen at − 80 °C.

#### Biochemical assays

The total protein content was determined using the method of Lowry et al*.*^50^. The activity of 17β–hydroxysteroid dehydrogenase activity was assayed according to the method of Bogovich and Payne^51^. The amount of reduced glutathione in the supernatant of testes tissues homogenate was determined using sulfosalicylic acid for protein precipitation and bis-(3-carboxy-4-nitrophenyl)-disulfide for color development^45^. Glutathione reductase activity was determined by measuring the oxidation of NADPH at 340 nm using the method of Suojanen et al.^53^. 1 nmole of NADPH oxidized/min/mg protein is one unit of enzyme activity.

Glutathione S-transferase [GST] activity was assessed according to the method of Lee et al.^54^. The conjugate of GSH with l-chloro-2, 4-dinitrobenzene (CDNB) was used detected at 340 nm using a double beam spectrophotometer. Under the assay conditions, a unit of enzyme activity is defined as the quantity of enzyme that catalyzes the production of 1 mmol of CDNB conjugate/mg protein/min. The molar extinction coefficient of 9.6 mM^−1^ cm^−1^ used in the calculations of GST activity. The activity of the glutathione peroxidase enzyme (GPx; EC. 1.11.1.9) was determined using the technique of Chiu et al.^55^. The enzyme source, 0.05 M Tris–HCl buffer (pH 7.6), 1.5 mM GSH, and cumene hydroperoxide added to a 1 ml reaction mixture and incubated for 5 min at 37 °C. The control sample was made without cumene hydroperoxides and incubated at 37 °C for 5 min in a separate tube. TCA (15%) used in both the control and test samples, while cumene hydroperoxide (0.1 ml) used alone in the control. Both tubes were incubated for 10 min at 37 °C before being centrifuged for 20 min at 3000 rpm. Catalase (CAT; EC1.11.1.6) activity was assayed in the supernatant fractions of testes homogenates according to the method of Luck^56^. The molar absorbance coefficient was used to calculate the amount of H_2_O_2_ decomposed over a certain time at a wavelength of 240 nm. Catalase activity is expressed as unite/mg protein.

Superoxide dismutase (SOD; EC 1.15.1.1) activity in the supernatant of testes homogenate was assayed by the method of Misra and Fridovich^57^. The generation of superoxide radicals by xanthine and xanthine oxidase, which react with nitro tetrazolium blue (NTB) to create formazan dye, used to estimate SOD activity. At 560 nm, the generated formazan dye was spectrophotometrically quantified. The degree of inhibition of this enzyme was measured in micromoles/minute/mg protein. Malondialdehyde (MDA), the lipid peroxidation end-product in testes, was quantified as thiobarbituric acid reactive substance (TBARS) using Tappel and Zalkin's technique^58^. At 532 nm, the color intensity of the reactants (MDA) measured. An extinction coefficient of 156,000 M^−1^ cm1 used for calculation of TBARS levels.

#### Western blotting and detection of the immobilized proteins

Pooled protein sample from each group (50 µg) was mixed with sample application buffer, then boiled for 3 min before being placed onto the gel. The gel was removed after the electrophoretic termination. To transfer proteins onto the nitrocellulose membrane, a V20-SDB semi-dry blotter was used. For 60 min, the current was set at 0.8 mA/cm2. The membranes were peeled off and rinsed twice with TBS for 15 min after the electrotransfer of protein bands was completed. After blocking for 1–2 h in a blocking buffer containing 5% non-fat dried milk, the membranes were washed twice with TBS for 15 min. Membranes were then incubated overnight with primary antibodies generated in goats for rabbits CYP21A1 and Anti-rabbit CYP19 and CYP11A1 raised in goats at a dilution of 1:1000 in 20 ml TBS, then washed twice with TBST (0.2 ml Tween 20/1 L TBS) for 20 min and then with TBS for 15 min. T-TBS for 20 min and TBS for 15 min were used to clean up the membranes twice. The membranes were then washed twice with T-TBS for 15 min after being incubated with secondary donkey anti-goat IgG-HRP at a dilution of 1:500 in 15 ml TBS. On the surface of the membrane, a combination of 1:1 luminal/enhancer and peroxide buffer was applied for 5 min as an immunodetection solution. The excess substrate was drained off the membrane's surface, which was then carefully wrapped in a transparent plastic sheet to protect it^59^. The fluorescent protein bands were then visualized using an x-ray film.

#### Histopathology

Small portions of testes of each group were preserved in 10% buffered formalin, then treated with standard grades of alcohol and xylol, embedded in paraffin, and sectioned at a thickness of 4–6 µm. The sections were stained with Hematoxylin and Eosin (H&E) and examined under a light microscope to examine the histopathological alterations in testes of various experimental groups (Leica DM 750)^60^.

### Statistical analyses

The mean, standard deviation, and standard error for each group were calculated using the SPSS 16 statistical software. The significance levels between groups were set at *P* < 0.05 and/or *P* < 0.001 using one-way ANOVA.

## Results

### Tramadol changed levels of steroid hormones

In the current investigation, testosterone levels in the serum of male rabbits were reduced as the duration of tramadol administration increased at 4, 8, and 12 weeks (Table 1). Pretreatment of rabbits with selenium and/or garlic for 2 h before tramadol administration was observed to raise and restore the reduced testosterone levels to normal levels (Table 1). On the other hand, pretreatment of rabbits with garlic extracts alone did not recover the tramadol-induced drop in estrogen levels compared to the control group (Table1). Interestingly, pretreatment of rabbits with selenium alone or in combination with garlic extract before administration of tramadol attenuated the decrease in estrogen levels compared to the control group (Table 1). Furthermore, motilities and sperm counts were reduced after treatment of rabbits for 4, 8, and 12 weeks with tramadol (Table 1). After pretreatment of rabbits with selenium, garlic extract, or a combination of the two, tramadol-induced sperm counts were restored to their normal levels (Table 1). Pretreatment of rabbits with selenium, garlic extract, or a combination of the two improved the lower motility of sperms produced by tramadol, but not to the normal levels (Table 1).

**Table 1:** Changes in levels of testosterone, estrogen, and semen paramters at 4, 8, and 12 weeks after pretreatment of male rabbits with selenium, garlic, and/or their combination before administration of tramadol by 2 h.

Weeks	Testosterone (ng/dl)					Estrogen (ng/dl)				
	Control	Tramadol	Tramadol + Se	Tramadol + garlic	Tramadol + Garlic + Se	Control	Tramadol	Tramadol + Se	Tramadol + garlic	Tramadol + Garlic + Se
4	7.6^a^ ± 0.3	6.4^c^ ± 0.2	7^abc^ ± 0.3	6.6^bc^ ± 0.5	7.2^ab^ ± 0.1	6.9^c^ ± 0.2	6.3^a^ ± 0.1	6.94^c^ ± 0.2	6.5 ^b^ ± 0.2	7.0^c^ ± 0.2
8	7.5^a^ ± 0.3	5.7 ^b^ ± 0.1	7.4^a^ ± 0.4	6.9^a^ ± 0.4	7.2^a^ ± 0.1	7^c^ ± 0.2	5.99^a^ ± 0.2	6.9^c^ ± 0.1	6.4^ab^ ± 0.3	6.8^bc^ ± 0.2
12	7.6^ab^ ± 0.2	4.7^c^ ± 0.1	7.8^a^ ± 0.2	7.3^b^ ± 0.3	7.8^a^ ± 0.1	7.2^d^ ± 0.2	5.4^a^ ± 0.1	6.78^c^ ± 0.3	6.3^b^ ± 0.2	6.7^c^ ± 0.1
	**The volume of semen [Ejacule] (ml)**					**pH of semen**				
4	1.5^a^ ± 0.1	1.0^c^ ± 0.1	1.3^ab^ ± 0.1	1.1^c^ ± 0.08	1.2 ± 0.1^bc^	7.53^ab^ ± 0.05	7.52^b^ ± 0.02	7.58^ab^ ± 0.06	7.52^b^ ± 0.01	7.6 ± 0.02^b^
8	1.4^a^ ± 0.2	0.8^c^ ± 0.09	1.4^a^ ± 0.04	1.1^bc^ ± 0.1	1.3^ab^ ± 0.1	7.54^a^ ± 0.07	7.4^b^ ± 0.07	7.57^a^ ± 0.76	7.51^ab^ ± 0.01	7.6^a^ ± 0.07
12	1.5^ab^ ± 0.1	0.5^c^ ± 0.1	1.5^a^ ± 0.2	1.2^c^ ± 0.1	1.3^bc^ ± 0.1	7.55^a^ ± 0.06	7.3^b^ ± 0.06	7.56^a^ ± 0.03	7.4^a^ ± 0.01	7.5^a^ ± 0.03
	**Motility %**					**Sperm count (million/ml)**				
4	207.0^a^ ± 5.6	127.0^c^ ± 5.6	160.0^b^ ± 7.9	135.0^c^ ± 5.5	146.0^bc^ ± 6	9.8^a^ ± 0.10	8.8^d^ ± 0.09	9.4^bc^ ± 0.06	9.4^c^ ± 0.05	9.6^b^ ± 0.08
8	208.0^a^ ± 9.6	86.0^c^ ± 6.0	156.0^b^ ± 8.3	126.0^c^ ± 6.7	139.0^bc^ ± 8	9.8^a^ ± 0.09	7.7^d^ ± 0.03	9.5^b^ ± 0.05	9.4^b^ ± 0.06	9.7^a^ ± 0.04
12	222.0^a^ ± 5.0	67.0^e^ ± 2.2	156.0^b^ ± 8.3	106.0^d^ ± 6.8	127.0^c^ ± 5.6	9.8^a^ ± 0.07	6.5^d^ ± 0.02	9.8^bc^ ± 0.03	9.6^c^ ± 0.05	9.8^ab^ ± 0.10
	**Sperm morphology %**									
4	7.88^c^ ± 0.30	14.50^a^ ± 0.67	10.14^b^ ± 0.61	12.36^b^ ± 0.74	9.84^bc^ ± 0.46					
8	7.86^d^ ± 0.27	18.36^a^ ± 1.49	13.94^b^ ± 0.84	16.60^b^ ± 3.06	12.86^c^ ± 0.93					
12	7.76^d^ ± 0.17	24.44^a^ ± 1.12^a^	17.48^b^ ± 1.18	18.86^b^ ± 1.49	16.22^d^ ± 0.84					

All values were presented as the mean and standard error of five rabbits for each treatment.

^abc^Means with different superscript letters were stastiscially significants.

^abc^Means with the same superscript letter were not stastiscially significant.

The level of significance for the differences between means was set at *P* < 0.05.

The semen volumes [Ejaculates] were decreased with increasing the duration of tramadol therapy of rabbits at 4, 8, and 12 weeks (Table 1). The decrease in ejaculates caused by tramadol, on the other hand, was restored to normal levels after pretreatment of rabbits with selenium (Table 1). However, pretreatment of rabbits with garlic extracts only could not restore such decrease caused by tramadol compared to the control group (Table 1). Interestingly, combining selenium with garlic extract reduced the tramadol-induced drop in ejaculates to normal levels (Table 1). Selenium proved to be more effective than garlic extract alone in restoring the decrease in ejaculate volumes caused by tramadol compared to the control group (Table 1).

### Effect of Tramadol on the antioxidant enzyme activities

Furthermore, the levels of free radicals were measured as thiobarbituric acid reactive substances which were markedly increased in the testes of tramadol-treated rabbits (Table 2). In addition, all antioxidant enzyme activities, including superoxide dismutase [SOD], glutathione peroxidases [GPx], and catalase [CAT] enzymes, were significantly inhibited in the testes of tramadol-treated rabbits (Table 2). However, pretreatment of rabbits with selenium or selenium plus garlic before tramadol administration was found to restore the inhibited antioxidant enzyme activities to their normal levels (Table 2). However, pretreatment of rabbits with garlic alone before tramadol administration increased the activity of SOD and CAT but did not reach their normal levels (Table 2). The activities of glutathione S-transferase [GST], glutathione reductase [GR], and reduced glutathione [GSH] levels were significantly decreased in tramadol-treated rabbits for 12 weeks compared to the control group (Table 2). On the other hand, pretreatment of rabbits with selenium, garlic, or their combination for 2 h before tramadol administration restored the tramadol-induced inhibition of GST, GR, and GSH to the normal levels (Table 2).

**Table 2:** Changes in the activity of 17β-hydroxysteroid dehydrogenase and antioxidant enzymes in testes of rabbits after pretreatment with selenium, garlic, or their combination for 2 h prior to tramadol administration.

Enzymes	Treatments				
	Control	Tramadol	Tramadol + Se	Tramadol + garlic	Tramadol + Garlic + Se
17β-hydroxysteroid dehydrogenase (Unit/ mg protein /min)	1.1561^d^ ± 0.08	0.75^a^ ± 0.02	1.5^b^ ± 0.02	1.4^b^ ± 0.02	1.8^a^ ± 0.04
Superoxide dismutase activity (U/mg protein)	149.0^a^ ± 3.6	70.0^d^ ± 2.1	128.0^b^ ± 0.1	91.0^c^ ± 2.7	111.0^b^ ± 3.1
Glutathione S-transferase (GST) (U/mg protein)	0.92^b^ ± 0.06	0.50^a^ ± 0.02	1.5^a^ ± 0.01	0.9^b^ ± 0.01	1.3^a^ ± 0.1
Glutathione reductase (µmol/g tissue)	85.20^b^ ± 1.90	44.0^d^ ± 1.25	96.9^a^ ± 1.21	69.9^c^ ± 1.39	75.0^c^ ± 0.79
Thiobarbituric acid reactive substances (µmol/g tissue)	1.83^b^ ± 0.02	3.8^a^ ± 0.23	0.87^c^ ± .032	1.3^bc^ ± 0.09	0.80^c^ ± .01
Glutathione peroxidase (GPx) (U/mg protein)	7.3^b^ ± 3.28	4.0^d^ ± 1.85	7.0^a^ ± 0.3	4.207^d^ ± 1.9	8.4^bc^ ± 3.75
Glutathione level (µmol GSH /g tissue)	4.4^ab^ ± 0.29	1.7^c^ ± 0.04	4.2^b^ ± 0.40	5.5^a^ ± 0.23	4.6^ab^ ± 0.24
Catalase activity (H_2_O_2_/mg protein/min)	66.7^a^ ± 1.20	43.2^c^ ± 1.71	86.2^a^ ± 3.33	54.6^bc^ ± 2.24	64.0^b^ ± 1.93

All values were presented as the mean and standard error of five rabbits.

^abc^Means with different superscript letters were stastiscially significants.

^abc^Means with the same superscript letter were not stastiscially significant.

The level of significance for the differences between means was set at *P* < 0.05.

### Effect of tramadol on the protein expression of cytochrome P450 isozymes and the activity of 17β-hydroxysteroid dehydrogenase

**Figure 1:**
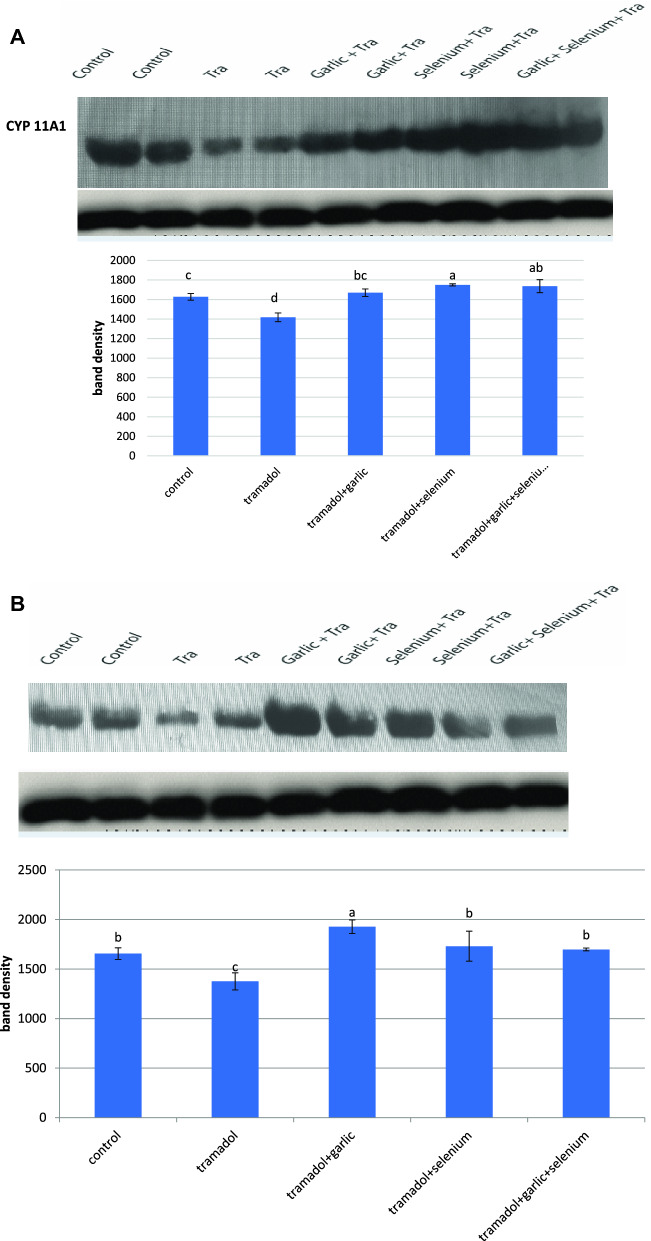

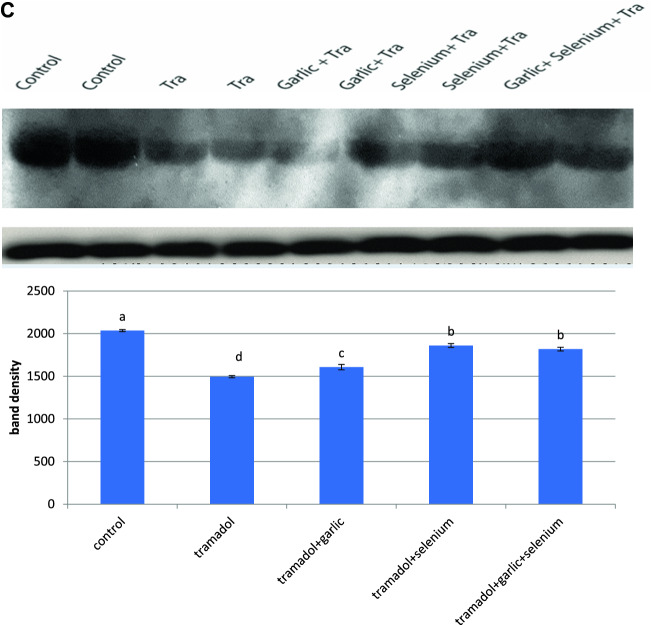
Western blot analysis showed the changes in CYP11A1 (**A**), CYP21A1 (**B**), CYP19 (**C**) protein expression after treatment of rabbits for 12 weeks with Tramadol (2 lanes), Tramadol + garlic (2 lanes), Tramadol + selenium (2 lanes), Tramadol + garlic + selenium (1 lane). (**B**) Quantification of the band density of each protein band was expressed in the histogram and columns with various letters are statistically significant [*P* < 0.05].

According to the findings of this study, the activity of 17-hydroxysteroid dehydrogenase was dramatically inhibited in the testes of rabbits given tramadol for 12 weeks (Table 2). However, pretreatment of rabbits for 2 h with selenium, garlic, and/or their combination before tramadol administration restored the decrease in 17-hydroxysteroid dehydrogenase activity caused by tramadol to the normal level (Table 2). Western immunoblotting data demonstrated that the protein expressions of CYP11A1, CYP21A2, and CYP19 were down-regulated in the testes of tramadol-treated rabbits (Fig. 1A,B,C). Pretreatment of rabbits with either selenium or garlic increased and upregulated the protein expression of CYP 11A1 levels above the normal level, whereas selenium was more efficient than garlic in restoring tramadol-induced down-regulation of CYP 11A1 (Fig. 1A). The downregulated protein expression of CYP21A1 was potentially upregulated after pretreatment of rabbits with garlic before tramadol administration (Fig. 1B). Garlic or garlic mixed with selenium was more effective than selenium alone in restoring and upregulating the tramadol-induced downregulation of the protein expression of CYP21A1 to its normal level (Fig. 1B). On the other hand, selenium was found to be more effective than garlic in restoring the tramadol-induced down-regulation of the protein expression of CYP19 (Fig. 1C).

### Histopathological examination of testicular tissues

**Figure 2:**
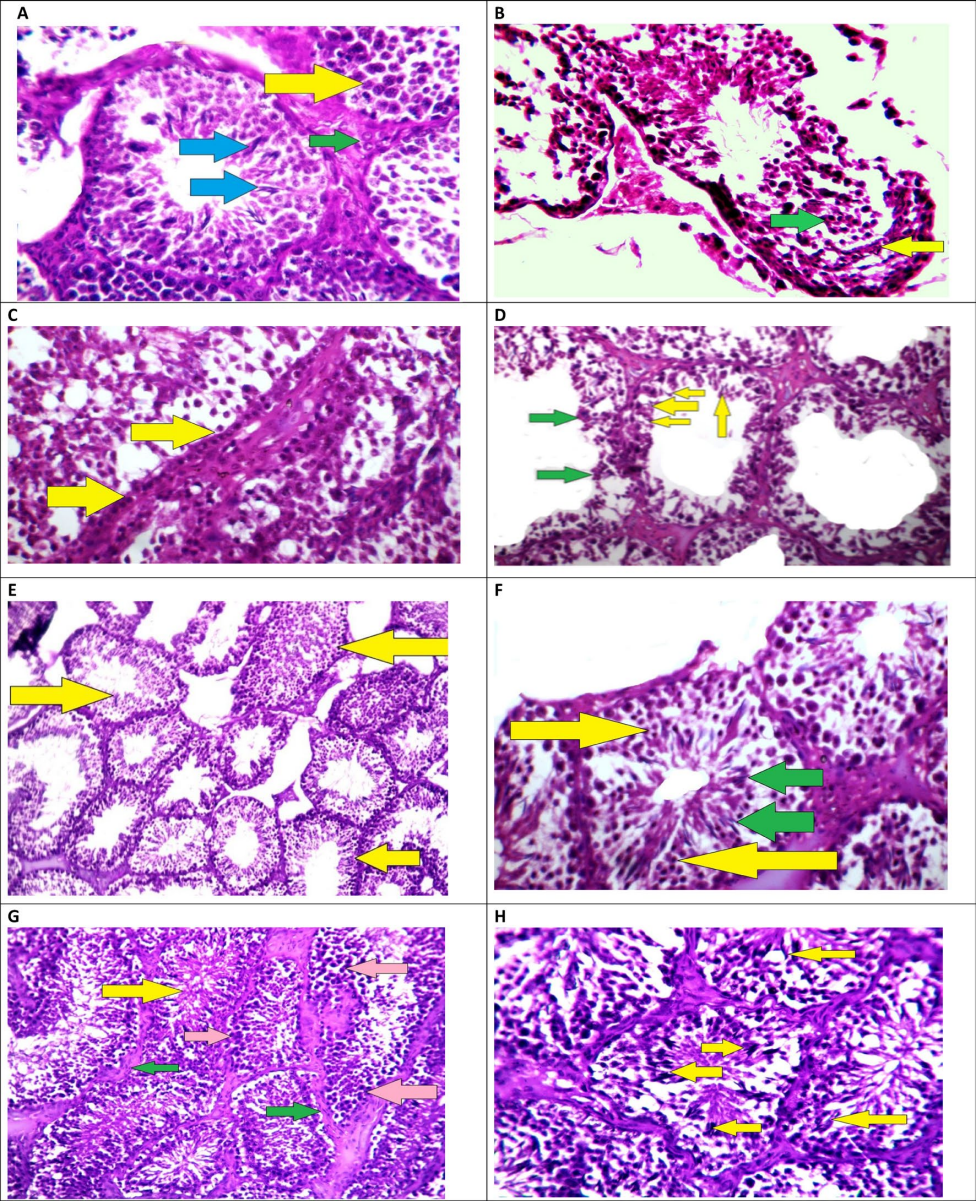
(**A)** Male rabbit testes from a healthy control group were examined histopathologically. Primary spermatocytes with condensed chromatin differentiated nuclei (yellow arrow), spermatozoa (blue arrows), and Leydig cells (cells that make testosterone) in a rabbit's testis (green arrow) (stain H&E ×400). (**B)** Male rabbit testes from the tramadol group showed degenerated spermatozoids near the basement membrane (yellow arrow), necrosed secondary spermatocytes with dark stain nuclei (green arrow), and disruption of the normal spermatogenic series are seen in the testis of a rabbit (StainH&EX200). (**C)** Testis of a rabbit pretreated with garlic before administration of tramadol showed high predominant of spermatogonium stem cells with undifferentiated nuclei (yellow arrows) (StainH&E ×200). (**D)** High magnification of C image showed sperm spermatozoids with abnormal bent shapes, some with preserved cytoplasm (yellow arrows), and degenerated necrosed cells (lost their nuclei; green arrows) (StainH&EX400). (**E)** Histopathological study of male rabbits testes of Tramadol and selenium group, in most of the testis tubules, histopathological examination revealed moderate to high improvement of the spermatogenic epithelium, good height and volume of spermatogenic epithelium (yellow arrows), and a few degenerated testis tubuli with low volume of spermatogenic epithelium (green arrows) (StainH&E ×200). (**F)** High power of E image showed high number of secondary spermatogenic cells (yellow arrows) and healthy spermatozoids (green arrows) (StainH&E × 400). (**G)** Histopathological analysis of male rabbit testes treated with Tramadol, garlic, or selenium showed a significant increase in the height and volume of the spermatogenic epithelium, a high percentage of spermatogenic stem cells (pink arrows), a high yield of healthy spermatozoid (yellow arrow), and a strong supporting interstitial tissue with myoid cells (green arrows) (stain H&E ×200). (**H)** High-resolution image reveals a high yield of spermatozoids (yellow arrows) and clear evidence of normal mitotic division, indicating a normal spermatogenic series (StainH&E ×400).

To verify the changes in the above biochemical data, a histological investigation of testes tissues was performed. Figure 2A showed the normal architecture of the control group's testes after they were given saline solution. Spermatogenic cells with considerable vacuolization and the presence of degraded immature spermatozoid after tramadol administration were found (Fig. 2B). The Tramadol-Garlic group had a higher number of spermatogenic stem cells sitting on the basement membrane, as well as some mitotic division abnormalities with aberrant chromatin, were present (Fig. 2C,D). Tramadol-selenium therapy caused an increase in the sperm numbers, as well as normal mitotic and meiosis divisions, were obtained (Fig. 2E,F). The Tramadol-garlic-selenium-treated rabbits showed a lot of sperm with healthy sperms, spermatogenic epithelium, and normal mitotic and meiotic divisions. Pretreatment of rabbits with selenium, and garlic either alone or in combination restored and alleviated the deleterious effects of tramadol in the architectures of testes (Fig. 2G,H).

## Discussion

Architectures of testis were found to be damaged as a result of exposure to toxic substances in both humans and rats^22,61^. Toxic substances have been identified to interact with hormone receptors and/or inhibit the activity of several enzymes involved in steroidogenesis, resulting in hormonal disruption^62^. Supporting these findings, it has been found that the testosterone and estrogen levels were decreased after chronic administration of tramadol to rabbits for 4, 8, and 12 weeks. In addition, the semen volumes decreased in tramadol-treated rabbits from 1 ml in the fourth week to 0.5 ml in the 12th week. Interestingly, pretreatment of rabbits with selenium and/or garlic before tramadol administration restored the decreased testosterone, and estrogen levels and volumes of semen compared to the control rabbits. Supporting our finding, the tramadol-treated female rats had lower estrogen levels in their serum, whereas the tramadol/vitamin C and tramadol/vitamin E treated groups had significantly higher levels^56^. In agreement with the previous studies, it has been found that opioids reduced serum testosterone levels in both rats, mice, and humans^1,63–67^. Histopathological investigation of tramadol-treated rabbit testes demonstrated vacuolization and the presence of disrupted immature spermatozoid. A healthy sperm, spermatogenic and normal mitotic and meiotic divisions were obtained in the tramadol-garlic-selenium treated group. Moreover, tramadol treatment reduced the number and the motility of sperms significantly in testes of rabbits, while pretreatment of rabbits with selenium and garlic restored such changes. In agreement with the present study, it has been found that tramadol therapy showed lower sperm counts and sperm abnormal forms^66–72^.

Cytochrome P450 isozymes [CYP21A1, CYP11A1, and CYP 19C] are involved in steroid hormone biosynthesis. In the present study, the downregulation of CYP21A1, CYP11A1, and CYP 19C protein expression and inhibition of 17-hydroxysteroid dehydrogenase in rabbit testes after tramadol administration may represent a unique mechanism resulting in reduced testosterone levels and sperm count. Furthermore, a decrease in plasma cholesterol levels [the substrate of testosterone synthesis] following tramadol therapy could be another plausible mechanism of sperm production and testosterone levels^10^. The present study showed that pretreatment of rabbits with garlic was more effective than selenium in restoring the down-regulated protein expression of CYP21A1 caused by tramadol over the normal levels. On the other hand, pretreatments of rabbits with selenium and/or selenium plus garlic were more effective than garlic only in restoring the tramadol-induced down-regulation of CYP19. Supporting our finding, pretreatment of rats with curcumin and/or gallic acid before tramadol administration recovered the down-regulated protein expression of CYP 2E1, 3A4, 2B1/2)^10^.

Seminal plasma contains several antioxidants that protect sperm from reactive oxygen species [ROS]. High amounts of ROS were reported to impair fertility by causing lipid oxidation, DNA damage, and sperm apoptosis^73,74^. Because spermatozoa have a low concentration of antioxidants, they are extremely susceptible to the adverse effects of ROS^10^. In addition, Oxidative stress is caused by high levels of ROS and is predominantly found in tissues with low antioxidant enzyme activity [SOD, CAT, GR, GPx, and GST]^10^. In the present study, tramadol therapy inhibited SOD, GSH, GST, CAT, GSR, and GPx activities and depleted glutathione levels, resulting in a rise in free radical levels and, as a result, increased oxidative stress in rabbits’ testes. In agreement with the present study, SOD, CAT, GR, GPx, and GST activities are inhibited in the livers of rats after tramadol treatment^10^. The mechanism of inhibition of antioxidant enzyme activities might be due to the interaction of tramadol or its metabolites with certain metal ions, such as selenium, that function as cofactors for antioxidant enzymes (SOD, GPx)^75^. Supporting this finding from the present study, when selenium was given to rabbits before tramadol therapy was found to increase the activities of SOD, GPx. Therefore, antioxidant treatments may be efficient therapeutic choices for dealing with the burden of oxidative stress-induced male infertility^70,74^. In agreement with our finding, pretreatment of rats with other antioxidants [Curcumin, Gallic acid, and/or Nigella sativa oil] before tramadol administration restored such inhibition in antioxidant enzyme activities^10,74,75^. Therefore, the low number of spermatozoa and testosterone levels in tramadol-treated rabbits could be due to depletion of glutathione levels and inhibition of antioxidant enzyme activity which leads to induction of free radical levels.

Garlic has an abundance of chemical compounds such as diallylthiosulfinate [allicin], alliin, S-allyl cysteines, thiacremonone, allyl sulfide, and diallyl sulfide^76^. Supporting our finding, it has been found that diallyl sulfide attenuated and improved the decrease in epididymal sperm count and motility, spermatogenesis score, serum testosterone, SOD, and GSH levels caused by lead in testes of rats^77^. Furthermore, diallyl sulfide enhanced testicular CYP19 expression significantly, which was downregulated after lead exposure^77^. Moreover, pre-treatments of rats with diallyl sulphide and/or aged garlic extract effectively reduced the testicular toxicity caused by cyclophosphamide and adriamycin, including decreased sperm count, epididymal sperm motility, and epididymal index, as well as increased histopathological alterations, decreased sperm count and abnormalities, low testosterone level, high MDA concentration, low GSH level, and decreased GSH-Px, CAT, and SOD activity in the test^78,79^.

It is concluded that tramadol therapy reduced testosterone levels, downregulated the protein expression of CYPs isozymes, inhibited antioxidant enzyme activity, and induced free radical levels. Such changes were reversed and alleviated in rabbits pretreated with garlic and/or selenium before tramadol administration. The findings of this study could point to a potential mechanism for lowering steroid hormone levels in tramadol-treated rabbits. Furthermore, it is advised to take garlic and/or selenium for at least 2 h before tramadol administration. Based on the outcomes of this investigation, other clinical trial studies may be suggested.

## Data Availability

All data generated or analyzed during this study are included in this published article [and its supplementary information files].

## References

[1] Ceccarelli I, De Padova AM, Fiorenzani P, Massafra C, Aloisi AM (2006). Single opioid administration modifies gonadal steroids in both the CNS and plasma of male rats. Neuroscience.

[2] Grond S, Sablotzki A (2004). Clinical pharmacology of tramadol. Clin. Pharmacokinet..

[3] Gillman PK (2005). Monoamine oxidase inhibitors, opioid analgesics and serotonin toxicity. Br. J. Anaesth..

[4] Miotto K, Cho AK, Khalil MA, Blanco K, Sasaki JD, Rawson R (2017). Trends in tramadol: Pharmacology, metabolism, and misuse. Anesth. Analg..

[5] de Moraes NV, Lauretti GR, Coelho EB, Godoy AL, Neves DV, Lanchote VL (2016). Impact of fraction unbound, CYP3A, and CYP2D6 in vivo activities, and other potential covariates to the clearance of tramadol enantiomers in patients with neuropathic pain. Fundam. Clin. Pharmacol..

[6] Tanaka H, Naito T, Sato H, Hiraide T, Yamada Y, Kawakami J (2018). Impact of CYP genotype and inflammatory markers on the plasma concentrations of tramadol and its demethylated metabolites and drug tolerability in cancer patients. Eur. J. Clin. Pharmacol..

[7] Saiz-Rodríguez M, Ochoa D, Román M, Zubiaur P, Koller D, Mejía G, Abad-Santos F (2020). Involvement of CYP2D6 and CYP2B6 on tramadol pharmacokinetics. Pharmacogenomics.

[8] Arafa MH, Atteia HH (2018). Genetic polymorphisms of cytochrome P450 2D6 (CYP2D6) are associated with long term tramadol treatment-induced oxidative damage and hepatotoxicity. Toxicol. Appl. Pharmacol..

[9] Raffa RB (2008). Basic pharmacology relevant to drug abuse assessment: tramadol as example. J. Clin. Pharm. Ther..

[10] Sheweita SA, Almasmari AA, El-Banna SG (2018). Tramadol-induced hepato- and nephrotoxicity in rats: Role of Curcumin and Gallic acid as antioxidants. PLoS ONE.

[11] Koohsari M, Ahangar N, Mohammadi E, Talebpour Amiri F, Shaki F (2020). Effects of tramadol administration on male reproductive toxicity in Wistar rats: The role of oxidative stress, mitochondrial dysfunction, apoptosis-related gene expression, and nuclear factor kappa B signalling. Bratisl. Lek. Listy.

[12] Koohsari M, Ahangar N, Mohammadi E, Shaki F (2020). Ameliorative effect of melatonin against reproductive toxicity of tramadol in rats via the regulation of oxidative stress, mitochondrial dysfunction, and apoptosis-related gene expression signaling pathway. Addict. Health..

[13] Abdellatief RB, Elgamal DA, Mohamed EE (2015). Effects of chronic tramadol administration on testicular tissue in rats: An experimental study. Andrologia.

[14] Hashim MA, El Rasheed AH, Ismail GAW, Awaad MI, El Habiby MM, Mohsen Ibrahim NM, Abdeen MS (2020). Sexual dysfunction in tramadol hydrochloride use disorder male patients: A case-control study. Int. Clin. Psychopharmacol..

[15] Rowe P, Comhaire FH, Hargreave T (1993). WHO Manual for the Standardized Investigation of the Infertile Couple.

[16] Alahmar AT (2017). Effect of vitamin C, vitamin E, zinc, selenium, and coenzyme Q10 in infertile men with idiopathic oligoasthenozoospermia. Int. J. Infertil. Fetal Med..

[17] World Health Organizaion [WHO] (1992). Laboratory Manual for the Examination of Human Semen and Sperm–Cervical Mucus Interaction.

[18] Sigman M, Lipshultz L, & Howards S. (1991) Evaluation of the subfertile male. In L. A. Lipshultz & S. S. Howards (Eds.), Infertility in the male. Chuchill Livingstone.

[19] Alahmar AT (2019). Role of oxidative stress in male infertility: An updated review. J. Hum. Reprod. Sci..

[20] Hull MG, Glazener CM, Kelly NJ, Conway DI, Foster PA, Hinton RA, Coulson C, Lambert PA, Watt EM, Desai KM (1985). Population study of causes, treatment, and outcome of infertility. Br. Med. J. (Clin. Res. Ed.).

[21] Gagnon C, Iwasaki A, De Lamirande E, Kovalski N (1991). Reactive oxygen species and human spermatozoa. Ann. N. Y. Acad. Sci..

[22] Sheweita SA, Tilmisany AM, Al-Sawaf H (2005). Mechanisms of male infertility: Role of antioxidants. Curr. Drug Metab..

[23] Dubin L, Amelar RD (1978). Varicocele. Urol. Clin. North Am..

[24] Saleh RA, Agarwal A, Sharma RK, Said TM, Sikka SC, Thomas AJ (2003). Evaluation of nuclear DNA damage in spermatozoa from infertile men with varicocele. Fertil. Steril..

[25] Vernet P, Aitken RJ, Drevet JR (2004). Antioxidant strategies in the epididymis. Mol. Cell Endocrinol..

[26] Hendin BN, Kolettis PN, Sharma RK, Thomas AJ, Agarwal A (1999). Varicocele is associated with elevated spermatozoal reactive oxygen species production and diminished seminal plasma antioxidant capacity. J. Urol..

[27] Mahfouz R, Sharma R, Thiyagarajan A, Kale V, Gupta S, Sabanegh E, Agarwal A (2010). Semen characteristics and sperm DNA fragmentation in infertile men with low and high levels of seminal reactive oxygen species. Fertil. Steril..

[28] Hanukoglu I (1992). Steroidogenic enzymes: structure, function, and role in regulation of steroid hormone biosynthesis. J. Steroid Biochem. Mol. Biol..

[29] Payne AH, Youngblood GL (1995). Regulation of expression of steroidogenic enzymes in Leydig cells. Biol. Reprod..

[30] Stocco DM, Chen W (1991). Presence of identical mitochondrial proteins in unstimulated constitutive steroid-producing R2C rat Leydig tumor and stimulated nonconstitutive steroid-producing MA-10 mouse Leydig tumor cells. Endocrinology.

[31] Ito K, Utsunomiya H, Suzuki T, Saitou S, Akahira J, Okamura K, Yaegashi N, Sasano H (2006). 17Beta-hydroxysteroid dehydrogenases in human endometrium and its disorders. Mol. Cell Endocrinol..

[32] Li Z, Luu-The V, Poisson-Paré D, Ouellet J, Li S, Labrie F, Pelletier G (2009). Expression of enzymes involved in synthesis and metabolism of estradiol in human breast as studied by immunocytochemistry and in situ hybridization. Histol. Histopathol..

[33] Hasegawa E, Nakagawa S, Sato M, Tachikawa E, Yamato S (2013). Effect of polyphenols on production of steroid hormones from human adrenocortical NCI-H295R cells. Biol. Pharm. Bull..

[34] Sagsak E, Aycan Z, Savas-Erdeve S, Keskin M, Cetinkaya S, Karaer K (2015). 17βHSD-3 enzyme deficiency due to novel mutations in the HSD17B3 gene diagnosed in a neonate. J. Pediatr. Endocrinol. Metab..

[35] Wang C, Ruan T, Liu J, He B, Zhou Q, Jiang G (2015). Perfluorooctyl iodide stimulates steroidogenesis in H295R cells via a cyclic adenosine monophosphate signaling pathway. Chem. Res. Toxicol..

[36] Geissler WM, Davis DL, Wu L, Bradshaw KD, Patel S, Mendonca BB, Elliston KO, Wilson JD, Russell DW, Andersson S (1994). Male pseudohermaphroditism caused by mutations of testicular 17 beta-hydroxysteroid dehydrogenase 3. Nat. Genet..

[37] Andersson S, Geissler WM, Wu L, Davis DL, Grumbach MM, New MI, Schwarz HP, Blethen SL, Mendonca BB, Bloise W, Witchel SF, Cutler GB, Griffin JE, Wilson JD, Russel DW (1996). Molecular genetics and pathophysiology of 17 beta-hydroxysteroid dehydrogenase 3 deficiency. J. Clin. Endocrinol. Metab..

[38] Chi XX, Chu XL, Zhang T, Cao LK (2019). Effect of genistein on the gene expressions of androgen generating key enzymes StAR, P450scc and CYP19 in rat ovary. Pol. J. Vet. Sci..

[39] Hu Y, Li D, Ma X, Liu R, Qi Y, Yuan C, Huang D (2021). Effects of 2,4-dichlorophenol exposure on zebrafish: Implications for the sex hormone synthesis. Aquat. Toxicol..

[40] El-Sharaky AS, Newairy AA, Badreldeen MM, Eweda SM, Sheweita SA (2007). Protective role of selenium against renal toxicity induced by cadmium in rats. Toxicology.

[41] Sheweita SA, El-Gabar MA, Bastawy M (2001). Carbon tetrachloride changes the activity of cytochrome P450 system in the liver of male rats: role of antioxidants. Toxicology.

[42] Barbosa J, Faria J, Leal S, Afonso LP, Lobo J, Queirós O, Moreira R, Carvalho F, Dinis-Oliveira RJ (2017). Acute administration of tramadol and tapentadol at effective analgesic and maximum tolerated doses causes hepato- and nephrotoxic effects in Wistar rats. Toxicology.

[43] Almodin CG, Minguetti-Câmara VC, Meister H, Ferreira JO, Franco RL, Cavalcante AA, Radaelli MR, Bahls AS, Moron AF, Murta CG (2004). Recovery of fertility after grafting of cryopreserved germinative tissue in female rabbits following radiotherapy. Hum. Reprod..

[44] Sheweita SA, Abd El-Gabar M, Bastawy M (2001). Carbon tetrachloride-induced changes in the activity of phase II drug-metabolizing enzyme in the liver of male rats: Role of antioxidants. Toxicology.

[45] Newairy AA, El-Sharaky AS, Badreldeen MM, Eweda SM, Sheweita SA (2007). The hepatoprotective effects of selenium against cadmium toxicity in rats. Toxicology.

[46] Atashfaraz E, Farokhi F, Najafi G (2013). Protective effect of ethyl pyruvate on epididymal sperm characteristics, oxidative stress and testosterone level in methotrexate treated mice. J. Reprod. Infertil..

[47] Freund M, Carol B (1964). Factors affecting haemocytometer counts of sperm concentration in human semen. J. Reprod. Fertil..

[48] Narayana K, Prashanthi N, Nayanatara A, Kumar HH, Abhilash K, Bairy KL (2005). Effects of methyl parathion (o, o-dimethyl o-4-nitrophenyl phosphorothioate) on rat sperm morphology and sperm count, but not fertility, are associated with decreased ascorbic acid level in the testis. Mutat. Res..

[49] Kong DY, Hao LR, Zhang L, Li QG, Zhou JH, Shi SZ, Zhu F, Geng YQ, Chen XM (2015). Comparison of two fluid solutions for resuscitation in a rabbit model of crush syndrome. Clin. Exp. Nephrol..

[50] Lowry OH, Rosebrough NJ, Farr AL, Randall RJ (1951). Protein measurement with the Folin phenol reagent. J. Biol. Chem..

[51] Bogovich K, Payne AH (1980). Purification of rat testicular microsomal 17-ketosteroid reductase. Evidence that 17-ketosteroid reductase and 17 beta-hydroxysteroid dehydrogenase are distinct enzymes. J. Biol. Chem..

[52] Mitchell JR, Jollow DJ, Potter WZ, Gillette JR, Brodie BB (1973). Acetaminophen-induced hepatic necrosis. IV. Protective role of glutathione. J. Pharmacol. Exp. Ther..

[53] Suojanen JN, Gay RJ, Hilf R (1980). Influence of estrogen on glutathione levels and glutathione-metabolizing enzymes in uteri and R3230AC mammary tumors of rats. Biochim. Biophys. Acta.

[54] Lee CY, Johnson L, Cox RH, McKinney JD, Lee SM (1981). Mouse liver glutathione S-transferases. Biochemical and immunological characterization. J. Biol. Chem..

[55] Chiu DT, Stults FH, Tappel AL (1976). Purification and properties of rat lung soluble glutathione peroxidase. Biochim. Biophys. Acta.

[56] Luck H. (1974) Estimation of catalase. In M. V. Bergmayer (Ed.), Method of enzymatic analysis (pp. 885). Academic Press.

[57] Misra HP, Fridovich I (1972). The role of superoxide anion in the autoxidation of epinephrine and a simple assay for superoxide dismutase. J. Biol. Chem..

[58] Tappel A, Zalkin H (1959). Inhibition of lipide peroxidation in mitochondria by vitamin E. Arch. Biochem. Biophys..

[59] Matsudaira P (1987). Sequence from picomole quantities of proteins electroblotted onto polyvinylidene difluoride membranes. J. Biol. Chem..

[60] Drury A, Wallington E (1980). Carleton's Histological Technique.

[61] Sheweita SA, El Banna YY, Balbaa M, Abdullah IA, Hassan HE (2017). N-nitrosamines induced infertility and hepatotoxicity in male rabbits. Environ. Toxicol..

[62] Chandra AK, Sengupta P, Goswami H, Sarkar M (2013). Effects of dietary magnesium on testicular histology, steroidogenesis, spermatogenesis and oxidative stress markers in adult rats. Indian J. Exp. Biol..

[63] Ukpanukpong RU, Eban LK, Adebiyi FD, Aigbadumah PO, Yusuff AA, Akpan UE (2019). Hormonal and electrolyte assessment of on the effect of garlic vitamin C and E in tramadol induced toxicity in female wistar rats. Eur. J. Biotechnol. Biosci..

[64] Ahmed MA, Kurkar A (2014). Effects of opioid (tramadol) treatment on testicular functions in adult male rats: The role of nitric oxide and oxidative stress. Clin. Exp. Pharmacol. Physiol..

[65] Farag AGA, Basha MA, Amin SA, Elnaidany NF, Elhelbawy NG, Mostafa MMT, Khodier SA, Ibrahem RA, Mahfouz RZ (2018). Tramadol (opioid) abuse is associated with a dose- and time-dependent poor sperm quality and hyperprolactinaemia in young men. Andrologia.

[66] Bassiony MM, Youssef UM, El-Gohari H (2020). Free testosterone and prolactin levels and sperm morphology and function among male patients with tramadol abuse: A case-control study. J. Clin. Psychopharmacol..

[67] Udefa AL, Beshel FN, Nwangwa JN, Mkpe ID, Ofuru OS, Sam-Ekpe VG, Stephen GI (2020). Vitamin E administration does not ameliorate tramadol-associated impairment of testicular function in Wistar rats. Andrologia.

[68] Ray JA, Kushnir MM, Meikle AW, Sindt JE, Strathmann FG (2017). An exploratory study evaluating the impact of opioid and non-opioid pain medications on serum/plasma free testosterone and free estradiol concentrations. Drug Test Anal..

[69] Koohsari M, Ahangar N, Mohammadi E, Shaki F (2020). Ameliorative effect of melatonin against reproductive toxicity of tramadol in rats via the regulation of oxidative stress, mitochondrial dysfunction, and apoptosis-related gene expression signaling pathway. Addict. Health.

[70] El Shal E, Selim MH (2015). The effect of tramadol treatment on rat testes and the possible protective role of selenium (light and electron microscopic study). Al-azhar Assiut Med. J..

[71] Ibrahim MA-L, Salah-Eldin A-E (2019). Chronic addiction to tramadol and withdrawal effect on the spermatogenesis and testicular tissues in adult male albino rats. Pharmacology.

[72] Takeshima T, Usui K, Mori K, Asai T, Yasuda K, Kuroda S, Yumura Y (2021). Oxidative stress and male infertility. Reprod. Med. Biol..

[73] Bisht S, Faiq M, Tolahunase M, Dada R (2017). Oxidative stress and male infertility. Nat. Rev. Urol..

[74] Abdel-Zaher AO, Abdel-Rahman MS, Elwasei FM (2011). Protective effect of Nigella sativa oil against tramadol-induced tolerance and dependence in mice: Role of nitric oxide and oxidative stress. Neurotoxicology.

[75] Kopalli SR, Cha KM, Cho JY, Kim SK, Koppula S (2022). Cordycepin mitigates spermatogenic and redox related expression in H_2_O_2_-exposed Leydig cells and regulates testicular oxidative apoptotic signalling in aged rats. Pharm Biol..

[76] Dorrigiv M, Zareiyan A, Hosseinzadeh H (2020). Garlic (*Allium sativum*) as an antidote or a protective agent against natural or chemical toxicities: A comprehensive update review. Phytother. Res..

[77] Hassan E, Kahilo K, Kamal T, El-Neweshy M, Hassan M (2019). Protective effect of diallyl sulfide against lead-mediated oxidative damage, apoptosis and down-regulation of CYP19 gene expression in rat testes. Life Sci..

[78] Kim SH, Lee IC, Baek HS, Moon C, Kim SH, Kim JC (2013). Protective effect of diallyl disulfide on cyclophosphamide-induced testicular toxicity in rats. Lab. Anim. Res..

[79] Nasr AY (2017). The impact of aged garlic extract on adriamycin-induced testicular changes in adult male Wistar rats. Acta Histochem..

