# Author Correction: Antioxidants (selenium and garlic) alleviated the adverse effects of tramadol on the reproductive system and oxidative stress markers in male rabbits

**DOI:** 10.1038/s41598-022-21203-6

**Published:** 2022-10-07

**Authors:** Salah A. Sheweita, Yassmin A. El‑dafrawi, Osama A. El‑ghalid, Alaa A. Ghoneim, Ahmed Wahid

**Affiliations:** 1 Department of Clinical Biochemistry, Faculty of Medicine, King Khalid University, P.O.Box: 960, Abha, 61421 Kingdom of Saudi Arabia; 2 Department of Biotechnology, Institute of Graduate Studies and Research, University of Alexandria, Alexandria, Egypt; 3 Poultry Physiology Department, Faculty of Agriculture, University of Alexandria, Alexandria, Egypt; 4 Department of Anaesthesia and Pain Management, Medical Research Institute, Alexandra University, Alexandria, Egypt; 5 Department of Pharmaceutical Biochemistry, Faculty of Pharmacy, Alexandria University, Alexandria, Egypt

Correction to: *Scientific Reports* 10.1038/s41598-022-16862-4, published online 17 August 2022

In the original version of this Article, Affiliation 3 and 4 were not listed in the correct order. The correct affiliations are listed below:

Affiliation 3:

Poultry Physiology Department, Faculty of Agriculture, University of Alexandria, Alexandria, Egypt

Affiliation 4:

Department of Anaesthesia and Pain Management, Medical Research Institute, Alexandra University, Alexandria, Egypt.

In addition, Salah A. Sheweita, Yassmin A. El-dafrawi and Ahmed Wahid were incorrectly affiliated with ‘Poultry Physiology Department, Faculty of Agriculture, University of Alexandria, Alexandria, Egypt’.

Alaa A. Ghoneim was incorrectly affiliated with ‘Department of Biotechnology, Institute of Graduate Studies and Research, University of Alexandria, Alexandria, Egypt’.

The original Article has been corrected.

